# The Mitochondrial Genes *BAK1*, *FIS1* and *SFN* are Linked with Alterations in Mitochondrial Membrane Potential in Barrett’s Esophagus

**DOI:** 10.3390/ijms19113483

**Published:** 2018-11-06

**Authors:** James J. Phelan, Finbar MacCarthy, Dermot O’Toole, Narayanasamy Ravi, John V. Reynolds, Jacintha O’Sullivan

**Affiliations:** 1 Trinity Translational Medicine Institute, Department of Surgery, St. James’s Hospital, D08W9RT Dublin, Ireland; phelanj1@tcd.ie (J.J.P.); ravisec@stjames.ie (N.R.); reynoldsjv@stjames.ie (J.V.R.); 2 Department of Clinical Medicine, Trinity Translational Medicine Institute, Trinity College Dublin and St. James’s Hospital, D08W9RT Dublin, Ireland; finbarmaccarthy@gmail.com (F.M.); otooled1@tcd.ie (D.O.)

**Keywords:** Barrett’s metaplasia, esophageal cancer, mitochondrial genes, mitochondrial dysfunction, mitochondrial membrane potential.

## Abstract

Barrett’s esophagus and esophageal cancer lack prognostic markers that allow the tailoring of personalized medicine and biomarkers with potential to provide insight into treatment response. This study aims to characterize mitochondrial function across the metaplasia-dysplasia-adenocarcinoma disease sequence in Barrett’s esophagus and examines the functional effect of manipulating mitochondrial genes. Mitochondrial genes of interest were validated in in vitro cell lines across the metaplasia (QH), dysplasia (GO) and adenocarcinoma (OE33) sequence and in in vivo patient tissue samples. These genes were subsequently knocked down in QH and OE33 cells and the functional effect of siRNA-induced knockdown on reactive oxygen species production, mitochondrial mass, mitochondrial membrane potential and cellular metabolism was investigated. Three global mitochondrial genes (*BAK1*, *FIS1* and *SFN*) were differentially altered across the in vivo Barrett’s disease sequence. We also demonstrate that knockdown of *BAK1*, *FIS1* and *SFN* in vitro resulted in significant alterations in mitochondrial membrane potential; however, no differences in reactive oxygen species or mitochondrial mass were observed. Furthermore, knockdown of these genes in esophageal adenocarcinoma cells significantly altered cellular metabolism. In conclusion, we found that differential expression of *BAK1*, *FIS1*, and *SFN* were altered across the Barrett’s disease sequence and manipulation of these genes elicited significant effects on mitochondrial membrane potential.

## 1. Introduction

The clinical management of Barrett’s esophagus has highlighted the need for accurate predictors of disease progression to esophageal cancer. The prognosis for individuals with esophageal adenocarcinoma (OAC) still remains inadequate with a survival rate of 9–15%; however, some cancer centers report survival rates between 35–50% [1,2]. Reliable prognostic markers would allow the tailoring of personalized medicine, influence personalized treatment regimens for individual patients and may prospectively determine if patients who have subsequently progressed to OAC would benefit from treatments. In the last decade, genomic instability profiles have displayed potential in the early diagnosis, prognosis and treatment response of various cancers; however, the role of mitochondrial instability and dysfunction still remains unclear [3,4,5]. 

The mitochondria have been implicated in cancer since the nineteenth century [6]. Early studies investigating the role of mitochondria in tumor cells concluded that the mitochondria were dysfunctional compartments; however, they are now known to play a central role in the cell and can even trigger disease progression [6]. Many studies also suggest that increases in mitochondrial activity are an important step for the generation of disseminated tumor cells, thereby contributing to metastases [7]. In normal cells, mitochondria play several roles in various anabolic and catabolic pathways including the maintenance of calcium homeostasis, the synthesis of ATP, the buffering of the redox potential within the cytosol and as a protagonist of apoptosis [6,8]. It is through these cellular mechanisms that mitochondria can support the initiation, transformation, differentiation and aggressive proliferation of tumor cells in both preneoplastic and neoplastic microenvironments.

Aerobic glycolysis has been shown to be the primary source of ATP in the majority of cancers; however, we have shown that both oxidative phosphorylation and glycolysis are both reprogrammed across the disease sequence in Barrett’s esophagus [9]. In addition, a marker of mitochondrial respiration, ATP5B, segregated Barrett’s patients who progressed to high-grade dysplasia (HGD)/OAC from non-progressors, highlighting the possible prognostic advantage of screening for mitochondrial markers in these preneoplastic Barrett’s patients [9]. Increased reactive oxygen species (ROS) are also implicated in the impairment of mitochondrial acetoacetyl-CoA thiolase in another inflammatory condition, ulcerative colitis [10]. Differential expression of the electron transport chain complexes is associated with disease progression in ulcerative colitis [11,12,13,14]. S100 calcium-binding protein P, carbamoyl-phosphate synthase 1, transcription factor Sp1, peroxisome proliferator-activated receptor-gamma coactivator-1α and c-myc are other mitochondrial associated proteins known to be involved in neoplastic progression in inflammatory disorders [14,15]. In addition, overexpression of mitochondrial translocator protein has demonstrated potential diagnostic and treatment value in inflammatory bowel disease [16]. There is a significant lack of assessment of these mitochondrial parameters in Barrett’s esophagus, another preneoplastic inflammatory condition.

The aim of this study was to examine mitochondrial gene expression and function across the disease sequence in vitro and in vivo in Barrett’s esophagus. We have identified genes associated with mitochondrial function that are differentially expressed across the metaplastic-dysplastic-OAC disease sequence both in vitro and in vivo. One of these genes was associated with mitochondrial fission (fission 1, or *FIS1*), one with apoptosis (bcl-2 homologous antagonist killer, or *BAK1*) and one with tumor suppression (stratifin, or *SFN*). We demonstrate through functional manipulation of *BAK1*, *FIS1* and *SFN*, significant alterations in mitochondrial membrane potential (MMP). Obtaining a better understanding of mitochondrial function in Barrett’s esophagus may reveal specific cellular mechanisms that promote neoplastic progression that could be exploited for improved therapeutic and prognostic applications.

## 2. Results

### 2.1. In Vitro Screening Using Human PCR Mitochondrial Gene Microarrays

To analyze the expression of 84 human genes associated with mitochondrial function across the disease sequence in vitro, fold expression of all target genes was normalized relative to the Barrett’s metaplastic cell line model, QH. Differentially expressed genes in our study were defined as those that changed by >4-fold and also those that have previously been linked with neoplastic progression in other diseases (the supplementary data file, S1 data, summarizes the relative expression of all 84 genes screened between QH and OE33 cell lines; also see this file for additional PCR microarray screen results between GO and QH and between GO and OE33 cells). Those that we choose for further analysis included the following three genes: *BAK1*, *FIS1* and *SFN*. These three gene targets identified were subsequently validated in vitro utilizing epithelial cell lines and in vivo using patient tissue samples and the levels of these genes manipulated to show a functional role.

**Figure 1:**
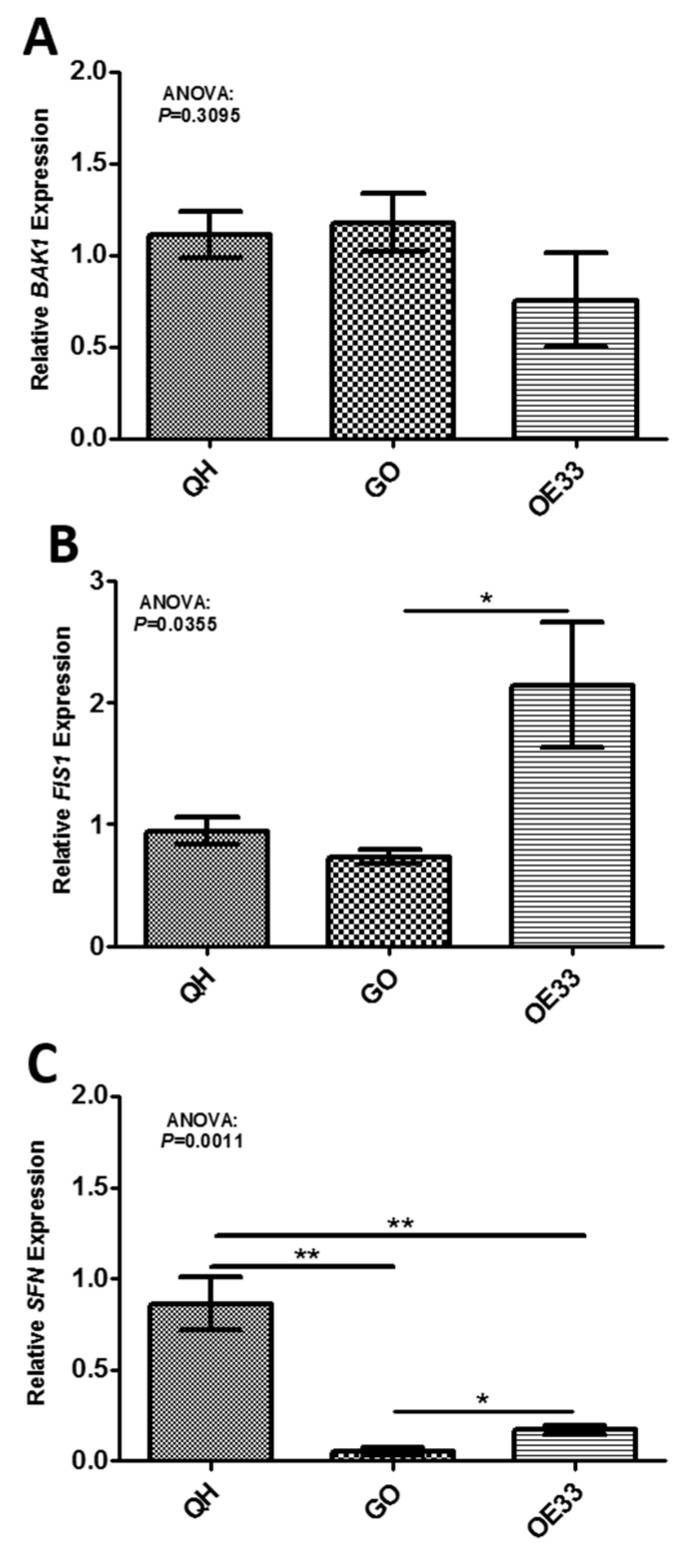
In vitro validation of global mitochondrial function gene targets found to be differentially expressed across the Barrett’s cell lines. (**A**) *BAK1* (*p* > 0.05), (**B**) *FIS1* (*p* < 0.05) and (**C**) *SFN* (*p* < 0.05) were differentially expressed between the in vitro Barrett’s cell lines (unpaired *t*-test; Bonferroni post-hoc test). One-way ANOVA was used to investigate differences across the in vitro Barrett’s sequence for *BAK1* (*p* = 0.3095), *FIS1* (*p* = 0.0355) and *SFN* (*p* = 0.0011). Bars denote mean ± SEM (*n* = 3). * *p* < 0.05 and ** *p* < 0.01.

Figure 1 shows the in vitro validation of the three mitochondrial gene targets chosen. While *BAK1* (Figure 1A) expression was not statistically altered across the in vitro Barrett’s sequence, *SFN* (*p* = 0.0011) expression significantly decreased between Barrett’s and OAC cell lines, but significantly increased between GO and OAC cell lines (Figure 1C). *FIS1* (*p* = 0.035) expression significantly increased across the in vitro Barrett’s sequence (Figure 1B). *FIS1* (*p* = 0.05) expression also significantly increased between GO and OE33 cell lines (Figure 1B).

### 2.2. In Vivo Validation of Gene Targets

**Figure 2:**
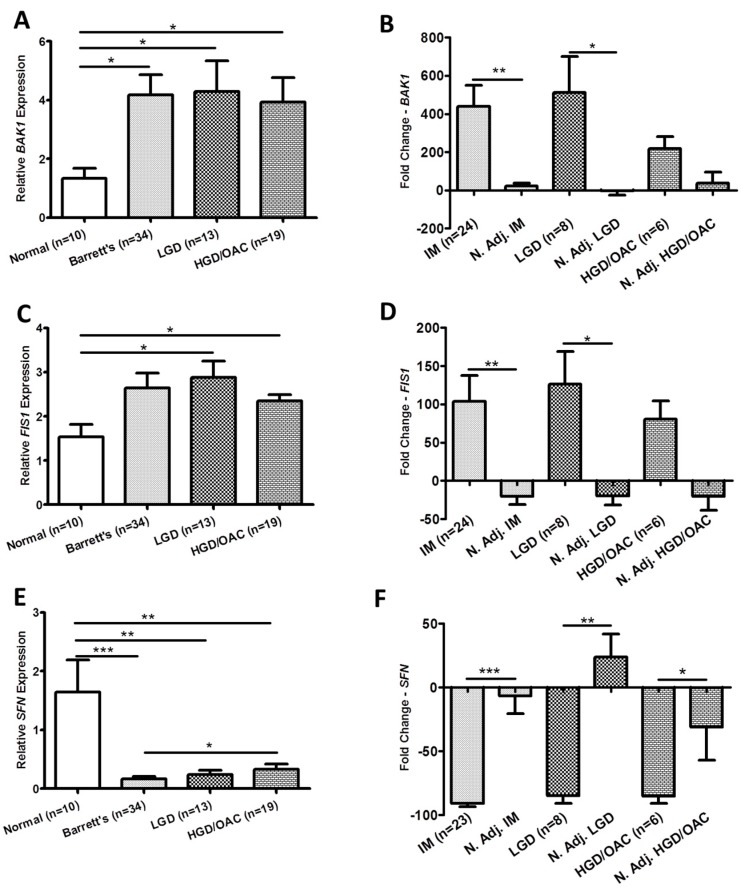
Global mitochondrial function gene expression across the disease sequence in diseased (**A**,**C**,**E**) versus matched normal adjacent (**B**,**D**,**F**) in vivo samples. (**A**) *BAK1* (*p* < 0.05), (**C**) *FIS1* (*p* < 0.05) and (**E**) *SFN* (*p* < 0.0001) were found to be differentially expressed between independent groups in the Barrett’s disease sequence (Mann Whitney U) (Dunns post-hoc test). Kruskal-Wallis tests were used to investigate differences across the in vitro Barrett’s sequence for *BAK1* (*p* = 0.037), *FIS1* (*p* = 0.108) and *SFN* (*p* < 0.0001). (**B**) *BAK1* (*p* < 0.01), (**D**) *FIS1* (*p* < 0.01) and (**F**) *SFN* (*p* < 0.001) were found to be differentially expressed across the Barrett’s disease sequence compared to matched normal adjacent samples (Wilcoxon Sign Rank). Bars denote mean ± SEM. * *p* < 0.05, ** *p* < 0.01 and *** *p* < 0.001.

We hypothesized that the biology between the epithelial cell lines and the patient tissues may be substantially different due to the intrinsic composition and complexity of the latter; therefore, we also needed to investigate the transcript levels of the same three genes in patient tissue samples. Figure 2 illustrates the expression of the three mitochondrial gene targets across the disease sequence in diseased and matched normal adjacent tissue samples. *BAK1* (Figure 2A) (*p* < 0.05), *FIS1* (Figure 2C) (*p* < 0.05) and *SFN* (Figure 2E) (*p* < 0.0001) were differentially expressed across the Barrett’s sequence. Field effect changes in gene expression of these targets in diseased versus matched normal adjacent biopsies was examined in a subset of patients where tissue was available. *BAK1* (Figure 2B) (*p* < 0.01), *FIS1* (Figure 2D) (*p* < 0.01) and *SFN* (Figure 2F) (*p* < 0.001) were differentially expressed across the Barrett’s disease sequence suggesting this effect was specific to the pathological diseased tissue (Barrett’s, LGD, HGD/OAC) compared to the surrounding matched mucosa. Due to the differential expression pattern of these three genes between pathological diseased tissue and the surrounding matched mucosa, the functional effect of *BAK1*, *FIS1* and *SFN* gene manipulation was further examined in vitro.

### 2.3. Functional Effect of BAK1, FIS1 and SFN siRNA Knockdown on Reactive Oxygen Species (ROS) Production, Mitochondrial Mass and Mitochondrial Membrane Potential (MMP) In Vitro

**Figure 3:**
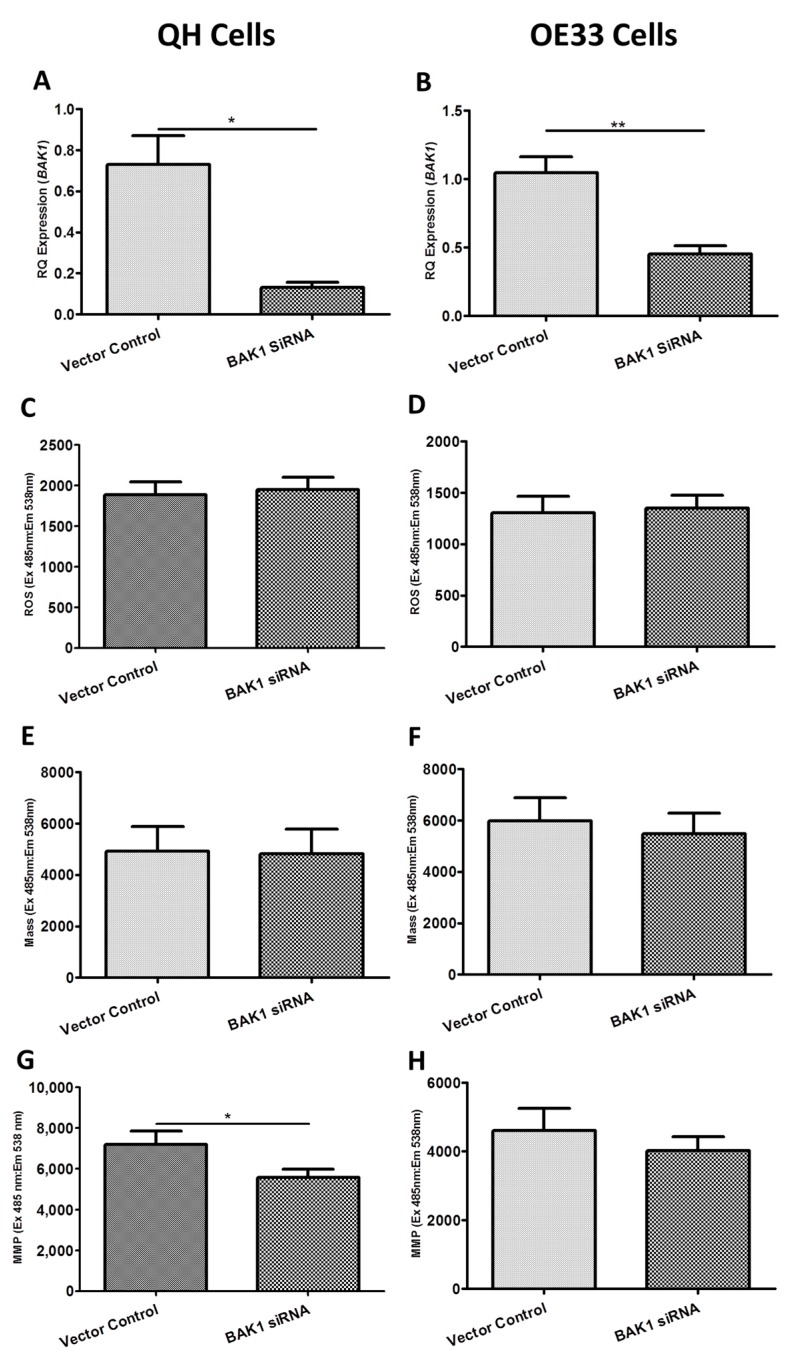
Functional effect of *BAK1* siRNA knockdown on reactive oxygen species (ROS) production, mitochondrial mass and mitochondrial membrane potential (MMP) in the QH (Barrett’s) and OE33 (adenocarcinoma) cell lines in vitro. (**A**) *BAK1* gene expression was significantly knocked down (81.9%) in the *BAK1*-siRNA treated QH cell line (*p* = 0.0191). (**B**) *BAK1* gene expression was significantly knocked down (56.9%) in the *BAK1*-siRNA treated OE33 cell line (*p* = 0.0030). (**C**) *BAK1* knockdown had no significant effect on ROS production in the QH (*p* > 0.05) or (**D**) OE33 cell lines (*p* = 0.5723). (**E**) *BAK1* knockdown had no significant effect on mitochondrial mass in QH (*p* = 0.8269) or (**F**) OE33 cell lines (*p* = 0.1114). (**G**) *BAK1* knockdown significantly decreased MMP in the QH cell line (*p* = 0.0454) (**H**) but had no significant effect on MMP in the OE33 cell line (*p* = 0.3080). Paired *t*-tests assessed statistical differences between vector control and siRNA treated QH (*n* = 4) and OE33 (*n* = 4) cells. Bars denote mean ± SEM. * *p* < 0.05 and ** *p* < 0.01.

To gain a functional understanding of *BAK1*, *FIS1* and *SFN*, these genes were knocked down in the Barrett’s and OAC cell lines in vitro as their expression was elevated in vivo from Barrett’s esophagus. *BAK1*, *FIS1* or *SFN* knockdown did not affect cell number in QH (Supplementary Figure S1A) or OE33 cells (Supplementary Figure S1B). Figure 3 shows the functional effect of *BAK1* siRNA knockdown on ROS production, mitochondrial mass and MMP in the Barrett’s and OAC cell lines. siRNA-induced knockdown of *BAK1* resulted in a significant reduction in *BAK1* expression in the QH (Figure 3A) (*p* = 0.019) and OE33 (Figure 3B) (*p* = 0.003) cell lines of 81.9% and 56.9%, respectively, compared to unscrambled control treated cells. Knockdown of *BAK1* in QH cells significantly decreased MMP (Figure 3G) (*p* = 0.045), while no effect was seen on ROS levels (Figure 3C) or mitochondrial mass (Figure 3E). In contrast, knockdown of *BAK1* had no effect on ROS levels (Figure 3D), mitochondrial mass (Figure 3F) or MMP (Figure 3H) in OE33 cells. Thus, changes induced by *BAK1* knockdown were specific to Barrett’s cells, which may be attributed to differences in the cellular biology between these two distinct preneoplastic (QH) and neoplastic (OE33) cell lines.

**Figure 4:**
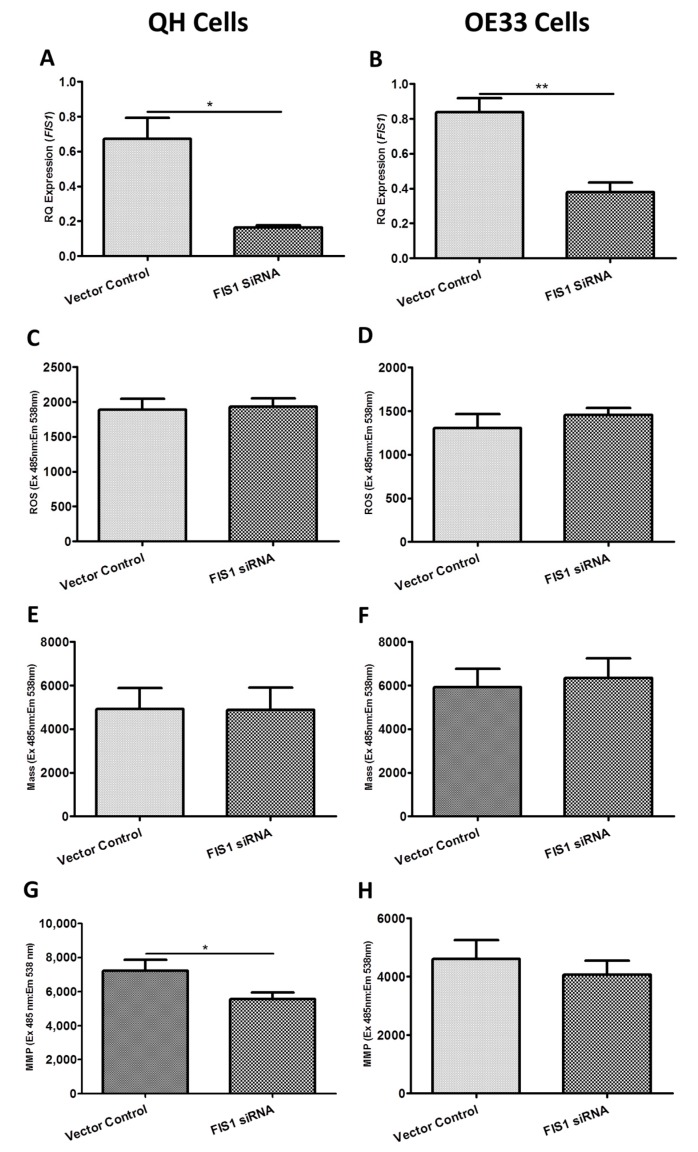
Functional effect of *FIS1* siRNA knockdown on reactive oxygen species (ROS) production, mitochondrial mass and mitochondrial membrane potential (MMP) in the QH (Barrett’s) and OE33 (adenocarcinoma) cell lines in vitro. (**A**) *FIS1* gene expression was significantly knocked down (75.9%) in the *FIS1*-siRNA treated QH cell line (*p* = 0.0242). (**B**) *FIS1* gene expression was significantly knocked down (54.8%) in the *FIS1*-siRNA treated OE33 cell line (*p* = 0.0037). (**C**) *FIS1* knockdown had no significant effect on ROS production in QH (*p* = 0.4112) and (**D**) OE33 cell lines (*p* = 0.2401). (**E**) *FIS1* knockdown had no significant effect on mitochondrial mass in the QH cell line (*p* = 0.9245) or in the (**F**) OE33 cell line (*p* > 0.05). (**G**) *FIS1* knockdown significantly decreased MMP in the QH cell line (*p* = 0.0190) but (**H**) had no significant effect on MMP in the OE33 cell line (*p* = 0.3124). Paired *t*-tests assessed statistical differences between vector control and siRNA treated QH (*n* = 4) and OE33 (*n* = 4) cells. Bars denote mean ± SEM. * *p* < 0.05 and ** *p* < 0.01.

Figure 4 shows the functional effect of *FIS1* siRNA knockdown on ROS production, mitochondrial mass and MMP in the Barrett’s and OAC cell lines. siRNA-induced knockdown of *FIS1* resulted in a significant reduction in *FIS1* expression in both the QH (Figure 4A) (*p* = 0.024) and OE33 (Figure 4B) (*p* = 0.004) cell lines of 75.9% and 54.8%, respectively, compared to unscrambled control treated cells. Analogous to the findings with *BAK1* in Figure 3, knockdown of *FIS1* significantly decreased MMP (Figure 4G) (*p* = 0.019) but did not alter ROS levels (Figure 4C) or mitochondrial mass (Figure 4E) in QH cells. Similarly, knockdown of *FIS1* did not significantly affect ROS levels (Figure 4D), mitochondrial mass (Figure 4F) or MMP (Figure 4H) in OE33 cells.

**Figure 5:**
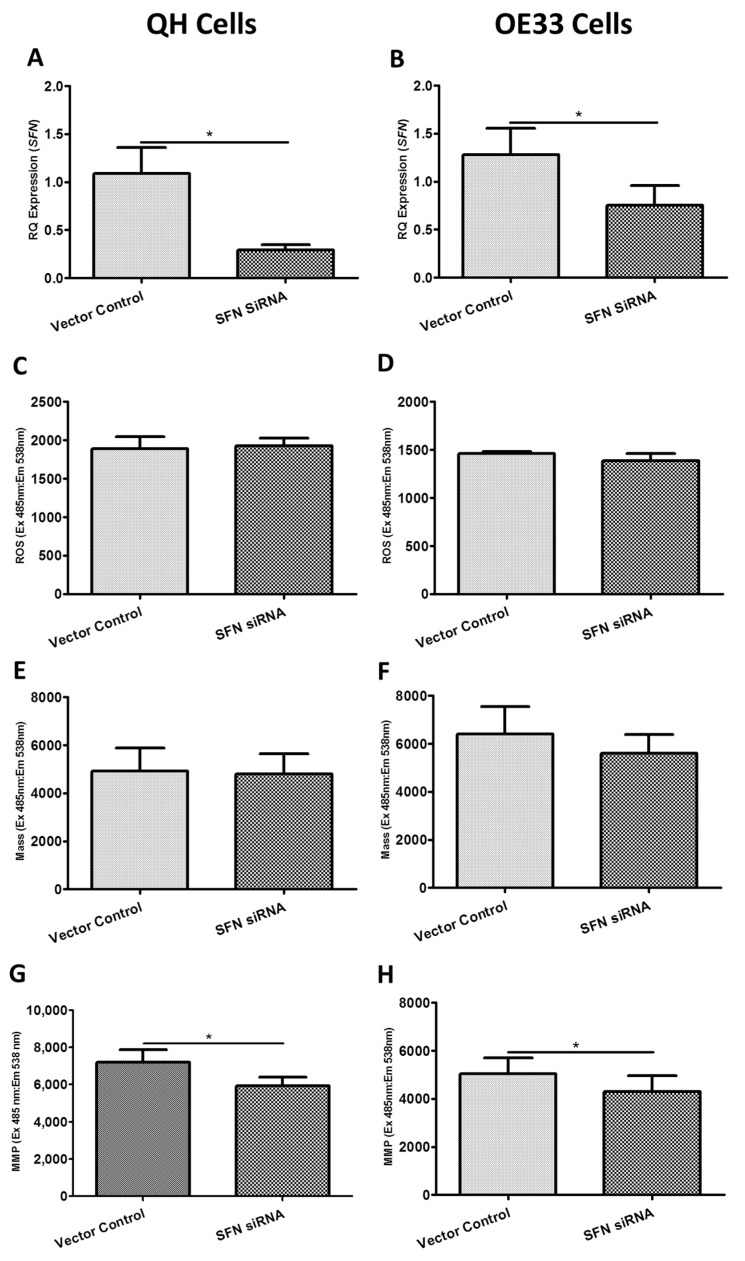
Functional effect of *SFN* siRNA knockdown on reactive oxygen species (ROS) production, mitochondrial mass and mitochondrial membrane potential (MMP) in the QH (Barrett’s) and OE33 (adenocarcinoma) cell lines in vitro. (**A**) *SFN* gene expression was significantly knocked down (73%) in the *SFN*-siRNA treated QH cell line (*p* = 0.0495). (**B**) *SFN* gene expression was significantly knocked down (41%) in the *SFN*-siRNA treated OE33 cell line (*p* = 0.0247). (**C**) *SFN* knockdown had no significant effect on ROS production in QH (*p* = 0.5619) or (**D**) OE33 cell lines (*p* = 0.3371). (**E**) *SFN* knockdown had no significant effect on mitochondrial mass in QH (*p* = 0.7858) or (**F**) OE33 cell lines (*p* = 0.1431). (**G**) *SFN* knockdown significantly decreased MMP in the QH (*p* = 0.049) and (**H**) OE33 cell lines (*p* = 0.0224). Paired *t*-tests assessed statistical differences between vector control and siRNA treated QH (*n* = 4) and OE33 (*n* = 3) cells. Bars denote mean ± SEM. * *p* < 0.05.

Figure 5 shows the functional effect of *SFN* siRNA knockdown on ROS production, mitochondrial mass and MMP in the Barrett’s and OAC cell lines. siRNA-induced knockdown of *SFN* resulted in a significant reduction in *SFN* expression in both the QH (Figure 5A) (*p* = 0.049) and OE33 (Figure 5B) (*p* = 0.025) cell lines of 73% and 41% respectively compared to unscrambled control treated cells. As with *BAK1* and *FIS1*, knockdown of *SFN* significantly decreased MMP (Figure 5G) (*p* = 0.049) but did not alter ROS levels (Figure 5C) or mitochondrial mass (Figure 5E) in QH cells. However, knockdown of *SFN* significantly decreased MMP (Figure 5H) (*p* = 0.022) without affecting ROS levels (Figure 5D) or mitochondrial mass (Figure 5F) in OE33 cells.

### 2.4. Assessing the Effect of BAK1, FIS1 and SFN siRNA Knockdown on Cellular Metabolism In Vitro

The effect of *BAK1*, *FIS1* and *SFN* knockdown on cellular metabolism was assessed. Oxygen consumption rate (OCR) and extracellular acidification rate (ECAR) were assessed as surrogate measures of oxidative phosphorylation and glycolysis respectively. *BAK1* knockdown did not significantly affect any aspect of metabolism examined in the QH or OE33 cells. Supplementary Figure S2 demonstrates the effect of *FIS1* and *SFN* knockdown on oligomycin-induced changes in ECAR (glycolysis) and baseline ECAR in QH and OE33 cells, respectively. Upon complex V inhibition with oligomycin, unscrambled control treated OE33 cells increased their glycolytic levels to compensate for the lack of oxidative phosphorylation activity; however, *FIS1* knockdown significantly decreased the ability of OE33 cells to increase glycolysis (Supplementary Figure S2B) (*P* = 0.006). Such observations were not seen in the QH cell line (Supplementary Figure S2A). Interestingly, *SFN* knockdown significantly decreased baseline levels of ECAR, or glycolysis, in OE33 cells (Supplementary Figure S2D) (*p* = 0.027), but had no effect in QH cells (Supplementary Figure S2C). No significant effect of *BAK1*, *FIS1* or *SFN* knockdown was observed on baseline OCR, ATP synthesis or proton leak in both cell lines (*p* > 0.05).

## 3. Discussion

Mitochondria play an important role in the initiation and progression of cancers [6]. As such, mitochondria are now considered a potential therapeutic target [17]. Identifying and targeting mitochondrial genes that modulate cellular function and metabolism may identify biomarkers of disease progression. Understanding how the mitochondria contribute to tumor growth may offer insight into the development of therapies which can negate neoplastic progression. We have shown that genes associated with mitochondrial function are altered across the normal-metaplasia-dysplasia-adenocarcinoma sequence of events in Barrett’s esophagus. These alterations in mitochondrial function may increase the risk of neoplastic progression from Barrett’s metaplasia to OAC. 

In this study, from a human PCR gene microarray, we chose to examine three genes associated with mitochondrial function differentially expressed between Barrett’s and OAC epithelial cells in vitro. One of these genes was associated with mitochondrial fission (fission 1, or *FIS1*), one with apoptosis (bcl-2 homologous antagonist killer, or *BAK1*) and one with tumor suppression (stratifin, or *SFN*). Compared to in vitro epithelial cells; however, in vivo samples are complex heterogeneous tissues composed of an assortment of specialized cells which may exhibit differential expression patterns. Therefore, we also characterized the expression of these mitochondrial genes in in vivo patient tissues.

We demonstrate that expression of the *BAK1* gene significantly increases across the disease sequence in vivo. This increase in expression was shown to be localized specifically in the Barrett’s tissue compared to the surrounding matched normal mucosa. Induction of apoptosis through *BAK1* activation has previously been shown to have a beneficial effect; specifically, overexpression of *BAK1* decreased in vitro growth, decreased cell cycle G0/G1 arrest and induced apoptosis in gastric cancer cells, suggesting *BAK1* may be a suitable therapeutic target for treating more proliferative tissue [18]. *BAK1* mediated apoptosis was also shown to correlate with caspase-3 activation and exert its apoptotic effect independent of p53 [18]. Infliximab-induced apoptosis in monocytes from patients with Crohn’s disease, and TRAIL-induced apoptosis in colon cancer cells has also been shown to be mediated by activation of BAK [19,20]. Conversely, one study investigating *BAK1* in tumor and non-tumor lesions in matched head and neck cancer patients found that loss of *BAK1* was associated with a non-tumor phenotype [21]. Moreover, although deletion of BAK significantly inhibited hepatocyte apoptosis in Mcl-1 knockout mice, deletion resulted in reduced incidences of liver cancer, reduced TNFα production, oxidative stress and oxidative DNA damage in non-cancerous livers [22]. BAK1 protein expression has also been shown to maintain a pro-oxidant state during periods of stress by regulating cytochrome c oxidase activity and mitochondrial respiration possibly indicating a novel anti-apoptotic role of BAK1 in conferring resistance to human leukemia and cervical cancer cells [23]. Therefore, expression of *BAK1* may play some role in Barrett’s patients by conferring resistance to potential cancer cells by regulating cytochrome c oxidase and mitochondrial energy metabolism, known to be differentially altered across the normal-metaplasia-dysplasia-adenocarcinoma disease sequence in Barrett’s esophagus [9].

We have shown that, *FIS1*, known to play important roles in apoptosis and mitochondrial fission, was shown to significantly increase across the disease sequence and this change was only seen in the Barrett’s tissue compared to matched surrounding mucosa. *FIS1* is a novel gene target associated with poor prognosis in pre-treatment patients with acute myeloid leukemia [24]. Conversely, it is also upregulated in patients with non-metastatic prostate cancer [25] and plays a role in cisplatin resistance in tongue squamous cell carcinoma with cisplatin sensitivity being restored upon knockdown of *FIS1* [26]. It is also speculated that increased levels of *FIS1* promotes mitophagy to eliminate defective mitochondria following cellular stress and perhaps supports functional oxidative phosphorylation known to be associated with progression to OAC in Barrett’s esophagus [9,27]. Therefore, increased *FIS1* in Barrett’s associated OAC may be indicative of mitochondrial dysfunction, enhanced mitophagy and oxidative stress.

Although highly expressed in normal adjacent tissues, we show an increase in *SFN* expression across the metaplastic-dysplastic-cancer sequence. Loss of *SFN* expression was shown to be specific to the Barrett’s tissue compared to the surrounding matched normal adjacent mucosa. Differential *SFN* expression patterns have been found in breast cancer cell lines and primary breast carcinomas [28]. Significant loss of *SFN* expression has also been documented in human hepatocellular carcinoma, lung cancer, oral cancer, prostate cancer, ovarian cancer and gliomas [29,30,31]. Some studies, however, have found *SFN* expression to be increased in some head and neck cancers and have demonstrated *SFN* as an independent prognostic marker for poor survival in colorectal cancer patients [30,32]. In esophageal squamous cell carcinoma (OSCC), downregulation of *SFN* has been shown to be associated with β-catenin expression, proliferation, invasion depth, lymph node metastasis and has been shown to have potential as a prognostic biomarker for OSCC [33,34,35]. Reduced *SFN* expression also functions as an independent prognostic factor for poor survival in patients with OAC and may be a potential target for more effective therapy and a potential predictor for the effect of chemoradiation therapy outcomes in patients with OAC [36,37]. Along with such evidence and the loss of *SFN* expression between metaplastic-dysplastic-adenocarcinoma tissue and normal adjacent tissues demonstrated in this study, *SFN* may have future promise as a prognostic biomarker in OAC.

**Figure 6:**
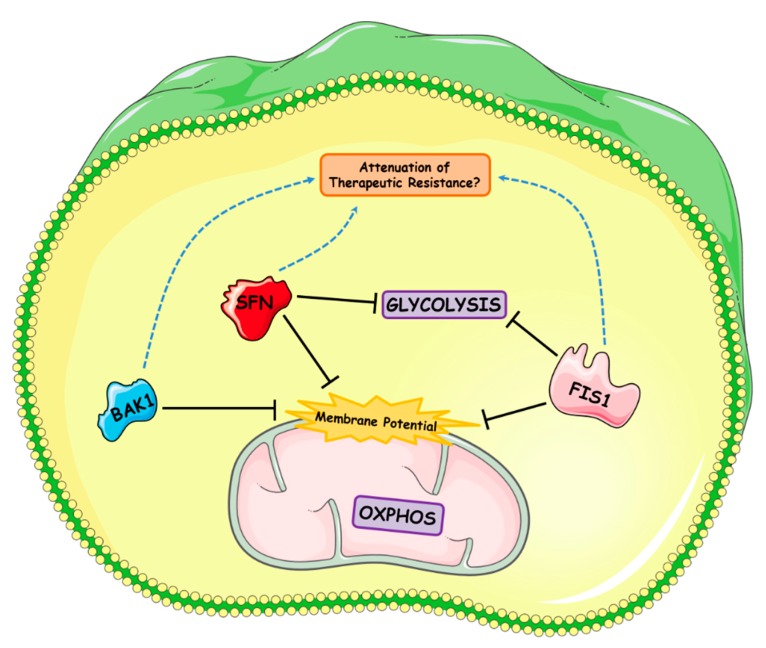
Summarizing the effect of *BAK1*, *FIS1* and *SFN* knockdown on mitochondrial function and cellular metabolism. Utilizing qPCR gene microarray screening, we screened 84 mitochondrial genes across the in vitro intestinal metaplasia-dysplasia-adenocarcinoma disease sequence and identified three genes as being differentially expressed: *BAK1*, *FIS1* and *SFN*. Moreover, upon further validation we found *BAK1*, *FIS1* and *SFN* were differentially expressed across the in vitro and in vivo intestinal metaplasia-dysplasia-adenocarcinoma disease sequence. These three genes were subsequently knocked down in vitro in Barrett’s (QH) and adenocarcinoma (OE33) cell lines to gain some insight into their potential role on mitochondrial function. We found that siRNA-induced knockdown of the three genes significantly decreased mitochondrial membrane potential in the Barrett’s cell line; thus, inhibition of *BAK1*, *FIS1* or *SFN* function may potentially make these cells more vulnerable to apoptosis in intestinal metaplasia or upon subsequent neoplastic progression. *SFN* knockdown also reduced baseline glycolysis in adenocarcinoma cells. Based on many previous studies, however, these three genes may also play an important part in conferring therapeutic resistance. Therefore, reagents that target these genes and their encoded products may help attenuate any harmful effects they encompass. Image produced with the aid of Servier Medical Art software (see copyright license at https://smart.servier.com).

To gain a better functional understanding of *BAK1*, *FIS1* and *SFN*, these genes were knocked down in the Barrett’s and OAC cell lines in vitro as their expression was elevated in vivo from Barrett’s esophagus. Moreover, their expression was shown to be specific to the Barrett’s tissue compared to the surrounding matched normal adjacent mucosa. ROS, mitochondrial mass, MMP and energy metabolism assays were examined upon siRNA-induced knockdown to assess the functional effect of manipulating each of these mitochondrial gene targets. Figure 6 summarizes the effect of *BAK1*, *FIS1* and *SFN* knockdown on mitochondrial function and cellular metabolism in Barrett’s and OAC cells.

In addition to demonstrating cell-specific effects between QH and OE33 cells as a result of siRNA treatment, we show that knockdown of *BAK1*, *FIS1* and *SFN* all resulted in significant decreases in MMP in Barrett’s cells. Little is known about the precise role of the MMP in preneoplastic tissue, but studies have documented elevated MMP in carcinomas compared to their matched normal controls; therefore, developing agents that target MMP, and thus apoptosis, is clinically appealing [38,39]. MMP is known to be significantly increased in neoplastic cells (CP-C) compared to Barrett’s cells (QH, or CP-A) further implicating mitochondria as one of the key factors in pre-malignant progression in Barrett’s esophagus [40]. Moreover, recent data implicates the MMP as being the key driver of the pro-inflammatory milieu in LPS-stimulated macrophages [41]. As delocalized lipophilic cations are concentrated into mitochondria by cells in response to negative transmembrane potentials, their selective accumulation within the mitochondria of cancer cells results in mitochondrial toxicity and a basis for selective cancer cell killing [38]. Such increases in MMP in the mitochondria of cancer cells can be targeted using positively charged ions that induce apoptosis [42]. Common consequences of apoptosis include decreases in MMP, release of pro-apoptotic proteins, increases in stress-induced ROS and arrest of cellular bioenergetics [39,43,44]. 

Furthermore, we found significant alterations in cellular energetics in siRNA treated OAC cells but not in Barrett’s cells. Loss of *FIS1* is known to negate mitochondrial fission thus promoting mitochondrial fusion. This loss of *FIS1* may favor a shift away from glycolysis to oxidative metabolism; however, the relationship between *FIS1* and cellular metabolism still needs to be examined in future studies. Furthermore, we demonstrate that loss of *SFN* in OAC cells is associated with lower levels of both MMP and glycolysis. As OAC is known to be associated with high levels of glycolysis, targeting *SFN* may potentially reduce the levels of glycolysis in these highly bioenergetics cells [9]. These observations suggest that therapies directed at these gene products, with a view to additionally influencing cellular energetics, may be a better therapeutic approach in the neoplastic setting.

Moreover, as decreased MMP can be an indicator of apoptosis, we investigated if knockdown of *BAK1*, *FIS1* or *SFN* had an effect on cell number [45]. We found that knockdown of these genes had no effect on cell number in both Barrett’s and OAC cells. The combined effect of *BAK1*, *FIS1* and *SFN* knockdown, however, may have a more substantial effect on mitochondrial function, cell viability and thus apoptosis. The extent of the knocking down of one of these genes alone may not reduce the MMP sufficiently to induce cellular apoptosis but may induce cellular alterations in mitochondrial function and cellular metabolism as we have demonstrated. Therefore, reagents that target gene products of *BAK1*, *FIS1* and S*FN* may need to be used in combination to induce increased levels of cell death. Based on previous studies investigating the role of *BAK1*, *FIS1*, and *SFN*, one primary function of these three genes in Barrett’s esophagus and in Barrett’s associated OAC may also be in conferring therapeutic resistance to cells; however, further studies are necessary to explore this relationship. Future studies also need to characterize BAK1, FIS1 and SFN protein expression profiles in Barrett’s and adenocarcinoma patients and correlate such expression profiles with surrogate markers associated with mitochondrial dysfunction to gain further insight into potential therapeutic or prognostic roles that these mitochondrial markers may encompass. 

We have demonstrated that global mitochondrial gene expression is differentially expressed across the normal-metaplastic-dysplastic-adenocarcinoma disease sequence in Barrett’s esophagus in vitro and in vivo. These mitochondrial changes may increase the risk of progression from Barrett’s esophagus to OAC. Examining whether these genes promote treatment resistance and oxidative stress, and determining the cellular mechanisms behind their functionality may provide some insight into screening for the levels of these biomarkers clinically in the future.

## 4. Materials and Methods 

### 4.1. Ethics

All methods within the study were carried out in accordance with the relevant guidelines and regulations. Ethical approval to conduct all aspects of this work was granted by the Adelaide and Meath Hospital (AMNCH), Tallaght, Dublin (REC 2011/04/05). All cases were prospectively recruited at our national referral center for upper GI malignancy. Written informed consent was obtained in accordance with local institutional ethical guidelines.

### 4.2. Metaplastic, Dysplastic and Adenocarcinogenic Cell Lines

QH (Barrett’s) and GO (dysplasia) cell lines, representing the Barrett’s and HGD stages of the Barrett’s disease sequence, were grown to 70% confluency in BEBM medium (2 mM glutamine, 10% FBS, 1% penicillin-streptomycin l-glutamine) supplemented with BEBM SingleQuots (2 mL BPE, 0.5 mL insulin, 0.5 mL HC, 0.5 mL GA-1000, 0.5 mL retinoic acid, 0.5 mL transferring, 0.5 mL triiodothyronine, 0.5 mL adrenaline and 0.5 mL hEGF per 500 mL media). OE33 cells, representing OAC, were grown to 70% confluency in RPMI medium (2 mM glutamine, 10% FBS, 1% penicillin-streptomycin l-glutamine). QH (or CP-A) and GO (or CP-B) cell lines were obtained from American Type Culture Collection (ATCC) (LGC Standards, Middlesex, UK). The OE33 cell line was sourced from the European Collection of Cell Cultures (Sailsbury, UK). Cell lines were authenticated and characterized by the suppliers and the suppliers use morphology, karyotyping and PCR-based approaches to confirm the identity of cell lines. Cell RNA extractions were subsequently performed using RNeasy Mini Kit (Qiagen, Hilden, Germany) following manufacturer’s instructions. RNA content and quality was quantified and assayed respectively and RNA reverse transcribed using the RT^2^ PCR array first strand kit (SABiosciences, Frederick, MD, USA).

### 4.3. Screening via qRT-PCR Microarray Analysis

The expression of 84 distinct mitochondrial genes across the in vitro sequence was quantified utilizing a human mitochondrial function microarray (Qiagen); the *B2M* gene was employed as the endogenous control gene. Following manufacturer’s instructions, PCR was performed using the RT^2^ Realtime SYBR Green PCR mix (SABiosciences) on a 7900HT Fast Realtime PCR Light-Cycler System (Applied Biosystems, Waltham, MA, USA). Data was analyzed utilizing the 2^−ΔΔ*C*t^ method. Significant genes of interest were defined as those that changed by >4-fold (with close duplicate *C*_t_ values) that have previously been linked with neoplastic progression which included genes that represent distinct elements of the mitochondrial function spectrum.

### 4.4. In Vitro qRT-PCR Validation of Gene Targets

QH, GO and OE33 cell lines were cultured and RNA extracted as described above. RNA was reverse transcribed using Bioscript enzyme (Bioline, London, UK). Gene primer probes for *BAK1*, *FIS1, SFN* and *18S* (Applied Biosystems) were purchased and real-time PCR was performed using Taqman mastermix (Applied Biosystems). Data was analyzed utilizing the 2^−ΔΔ*C*t^ method.

### 4.5. In Vivo qRT-PCR Validation of Gene Targets

The expression of *BAK1*, *FIS1* and *SFN* were analyzed in independent groups of patient tissues across the Barrett’s sequence. Realtime PCR was performed and the expression of *BAK1*, *FIS1* and *SFN* validated similarly across the diseased Barrett’s sequence in vivo utilizing normal squamous (*n* = 10), metaplasia (*n* = 34), LGD (*n* = 13), HGD (*n* = 12) and OAC (*n* = 8) cases. The median age of the patients was 61 years and there was a 1.52-fold male predominance. All patients attending with histologically confirmed Barrett’s esophagus or OAC (with a background of Barrett’s esophagus) were considered for inclusion. Patients previously treated for OAC, those who presented with another malignancy of any type and those who received ablative therapy were excluded.

White light and chromoendoscopy was employed for endoscopic examination in all cases (FICE (fujinon) or NBI (olympus)) (by DOT, NR & FMC). The Barrett’s segment was measured as per the Prague classification system and suspicious sites were biopsied using large capacity forceps, with mapping biopsies performed thereafter. Matched squamous samples were taken at least 5 cm superior to the proximal border of Barrett’s mucosa.

Normal control samples, demonstrating normal squamous mucosa, were taken from individuals attending for upper GI endoscopy without symptoms of gastroesophageal reflux disease (GORD) or other inflammatory etiology. Cancer tissue samples were taken from individuals undergoing assessment for OAC. At the time of endoscopy, all samples were placed in RNAlater. Barrett’s tissue was characterized by assessing the expression of cytokeratin 8 and villin by RT-PCR. Patient material was homogenized using a Tissue-Lyser for 5 min at a frequency of 25 pulses per second (Qiagen). RNA was subsequently extracted and reverse transcribed as per the manufacturer’s instructions (Bioline). RNA was then quantified (NanoDrop, Technologies, Wilmington, DE, USA). The quality of all RNA samples was additionally evaluated using the RNA Nano 6000 kit (Agilent technologies, Santa Clara, CA, USA) (undertaken by FM).

### 4.6. In Vitro siRNA Knockdown of Gene Targets

QH (Barrett’s) and OE33 (OAC) cell lines, representing intestinal metaplasia and adenocarcinoma respectively, were grown and cultured to 70% confluency as above. Both cell lines were seeded for mitochondrial and Seahorse assays at 15,000 cells per well (96 well format) and simultaneously reverse transfected with previously optimized 10 nM HLC purified 27mer Dicer-substrate siRNA duplexes specific to *BAK1*, *FIS1* and *SFN* with an adequate universal scrambled negative control siRNA duplex (Origene, Rockville, MD, USA). Moreover, QH and OE33 cells were treated with 10 μL and 20 μL of siTran 1.0 transfection reagent (Origene), respectively, per 10 nM siRNA treatment and were incubated at 37 °C in a CO_2_ incubator for 24 h prior to commencing mitochondrial and Seahorse assays. Additional cells were reverse transfected similarly to subsequently determine the percentage of knockdown efficiency through qRT-PCR. For each experimental assay, qRT-PCR was undertaken as described above.

### 4.7. Exploring the Functional Effect of siRNA Knockdown on Reactive Oxygen Species Production, Mitochondrial Mass and Mitochondrial Membrane Potential In Vitro

QH and OE33 cells were seeded at 15,000 cells per well in a 96-well cell culture plate, reverse transfected as described above and allowed to incubate for 24 h. Cell supernatant was removed and the cells were washed with 100 μL PBS/Mg^2+^ buffer (130 mM NaCl, 5 mM KCl, 1 mM Na^2^PO_4_, 1 mM CaCl_2_, 1 mM MgCl_2_ and 25 mM HEPES, pH 7.4) and subsequently incubated for 30 min at 37 °C in PBS/Mg^2+^ containing 5 µM 2′,7′-dichlorofluorescein (Invitrogen), 0.3 µM mitotracker (Invitrogen, California, USA) or 5 µM rhodamine 123 (Sigma, St. Louis, MO, USA) to assess reactive oxygen species production, mitochondrial mass and mitochondrial membrane potential respectively. 2′,7′-dichlorofluorescein and mitotracker were solubilized in DMSO, whereas rhodamine 123 was solubilized in ethanol. Cells were washed with 200 μL PBS/Mg^2+^ buffer, the buffer discarded, 100 μL fresh buffer added and fluorescence read with excitation and emission wavelengths of 485 nm and 538 nm respectively (Thermo Scientific, Fluoroskan Ascent FL, Waltham, MA, USA). All measurements were normalized to cell number using the crystal violet assay (Sigma) (9).

### 4.8. Characterizing the Metabolic Effect of siRNA Knockdown of BAK1, FIS1 and SFN Utilizing the Seahorse XF^E^24 Analyzer In Vitro

Oxidative phosphorylation and glycolysis, were measured by assaying oxygen consumption rate (OCR) and extracellular acidification rate (ECAR) respectively before and subsequent to treatment with oligomycin (2.5 µM, Sigma), trifluorocarbonylcyanide phenylhydrazone (FCCP) (2 µM, Sigma), antimycin-A (2 µM, Sigma) and 2-deoxy-glucoe (2-DG) (100 mM, Seahorse Biosciences, North Billerica, MA, USA) using the Seahorse XF24 analyzer (Seahorse Biosciences). QH and OE33 cells were seeded at 15,000 cells per well in a 24-well cell culture XF microplate (Seahorse Biosciences), reverse transfected as described above and allowed to incubate for 24 h. Cells were rinsed with assay medium (unbuffered DMEM supplemented with 10 mM glucose, 5 mM sodium pyruvate and 1 mM l-glutamine, pH 7.4) before incubation with assay medium for 1 h at 37 °C in a non-CO_2_ incubator. Three baseline OCR and ECAR measurements were obtained over 21 min before injection of specific metabolic inhibitors. Three OCR and ECAR measurements were obtained over 15 min following injection with oligomycin, FCCP, antimycin-A and 2-DG. Percentage non-mitochondrial respiration was calculated by expressing residual OCR post antimycin-A injection as a percentage of baseline OCR. Oligomycin-induced compensatory glycolysis was calculated by plotting ECAR as a percentage of baseline ECAR post oligomycin injection. The experiment was repeated four times (*n* = 4), with at least three technical replicates. All measurements were normalized to cell number using the crystal violet assay.

### 4.9. Statistical Analysis

Data was analyzed using Graph Pad Prism software (Graph Pad Prism, San Diego, CA, USA). One-way ANOVA was used to investigate differences in *BAK1*, *FIS1* and *SFN* expression across the in vitro Barrett’s sequence (Bonferroni post-hoc test; unpaired *t*-test). qRT-PCR in vivo data was normalized using the 2^−ΔΔ*C*t^ method and statistically analyzed using a Kruskal-Wallis test (Dunns post-hoc test; Mann-Whitney U). qRT-PCR target genes were statistically analyzed (Wilcoxon Sign Rank) in matched patient samples to explore if observed gene expression changes were an effect seen in the Barrett’s tissue compared to the surrounding mucosa. Student’s paired *t*-tests were utilized to compare differences in vector control and siRNA treated groups for all in vitro mitochondrial and Seahorse assays. Differences of *p* < 0.05 (*), *p* < 0.01 (**) and *p* < 0.001 (***) were considered statistically significant.

## Supplementary Materials

Supplementary materials can be found at http://www.mdpi.com/1422-0067/19/11/3483/s1.
