# Supplementary material for: The Mitochondrial Genes BAK1, FIS1 and SFN are Linked with Alterations in Mitochondrial Membrane Potential in Barrett’s Esophagus
Source: Int J Mol Sci. 2018 Nov 6;19(11):3483. doi: 10.3390/ijms19113483 (PMC6275077; doi:10.3390/ijms19113483)
Supplement: Supplementary file 1 [file ijms-19-03483-s001.zip › Titles and legends to supplementary figures 1 and 2.pdf]

Titles and legends to supplementary figures 1 & 2

Supplementary Figure 1. Assessing the effect of *BAK1, FIS1* and *SFN* siRNA knockdown on cell number in QH and OE33 cells in vitro using a crystal violet assay. (**A**) Knockdown of *BAK1, FIS1* and *SFN* had no significant effect on cell number in QH cells (P = 0.1948). (**B**) Knockdown of *BAK1, FIS1* and *SFN* had no significant effect on cell number in OE33 cells (P = 0.6874) (One way ANOVA; Dunnett's multiple comparison post-hoc test). Bars denote mean ± SEM.

Supplementary Figure 2. Investigating the metabolic effect of siRNA-induced knockdown of *FIS1* and SFN in the QH (Barrett's) and OE33 (adenocarcinoma) cell lines in vitro. (**A**) *FIS1* knockdown did not affect oligomycin-induced compensatory glycolysis in the QH cell line (P = 0.3230). (**B**) *FIS1* knockdown significantly decreased oligomycin-induced compensatory glycolysis in the OE33 cell line (P = 0.0065). (**C**) *SFN* knockdown did not affect baseline ECAR (glycolysis) in the QH cell line (P = 0.1034). (**D**) *SFN* knockdown significantly decreased ECAR (glycolysis) glycolysis in the OE33 cell line (P = 0.0271). Paired t-tests assessed statistical differences between vector control and siRNA treated QH (n = 4–5) and OE33 (n = 4–5) cells. Bars denote mean ± SEM. * $P<0.05$ and ** $P<0.01$.
