# Supplementary figures and images for: The Mitochondrial Genes BAK1, FIS1 and SFN are Linked with Alterations in Mitochondrial Membrane Potential in Barrett’s Esophagus
Source: Int J Mol Sci. 2018 Nov 6;19(11):3483. doi: 10.3390/ijms19113483 (PMC6275077; doi:10.3390/ijms19113483)

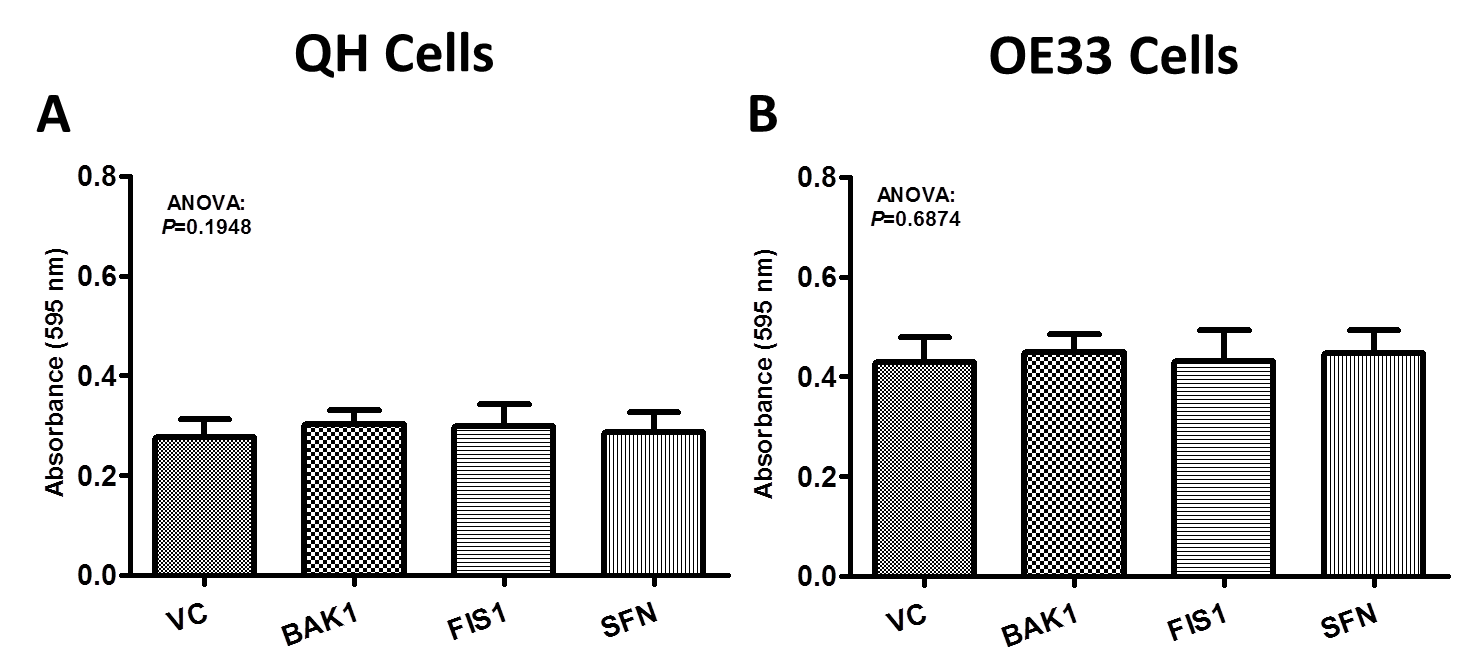

Supplement: Supplementary file 1 [file ijms-19-03483-s001.zip › Supplementary Figure 1.tif]

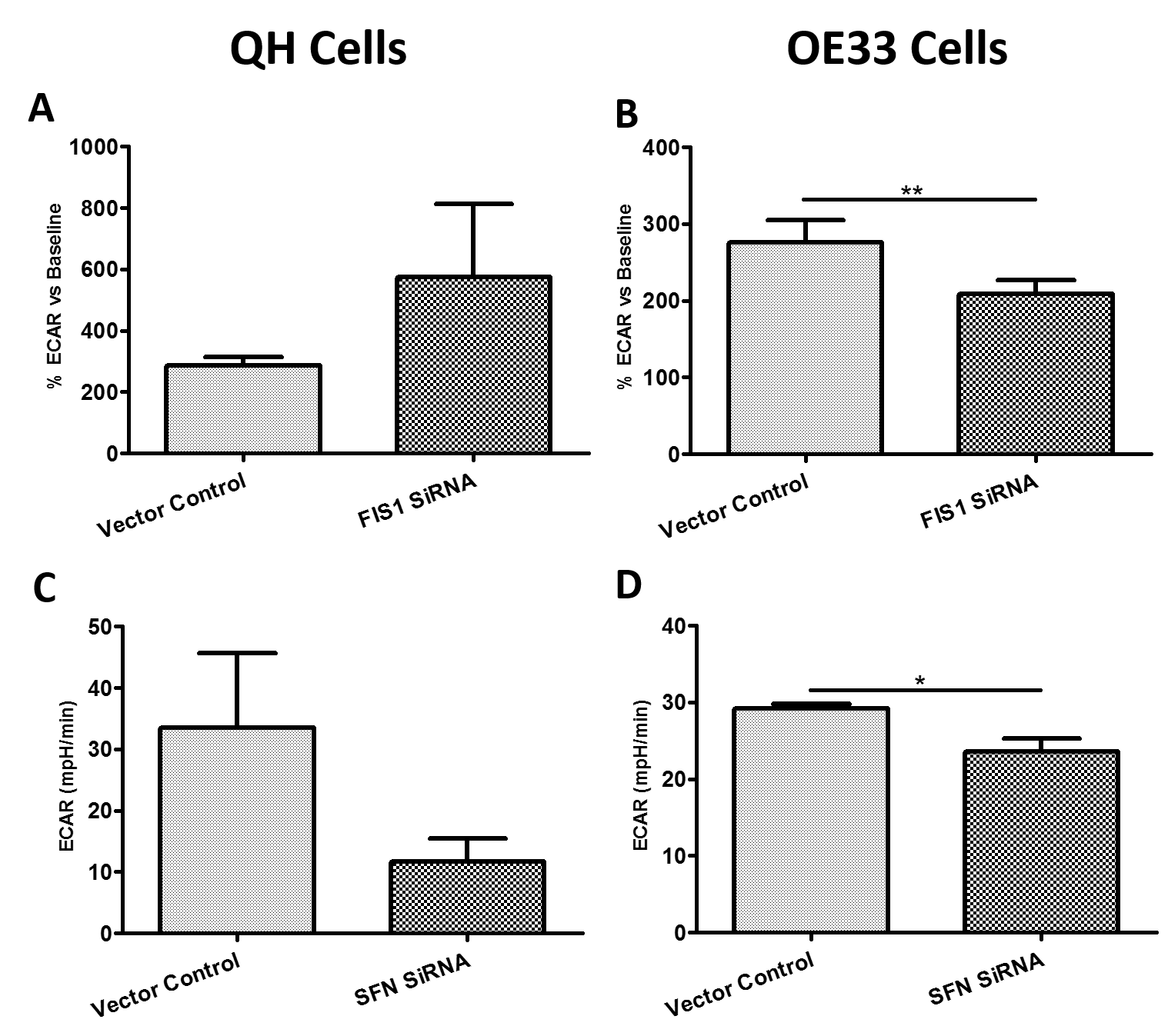

Supplement: Supplementary file 1 [file ijms-19-03483-s001.zip › Supplementary Figure 2.tif]
